# Age‐adjusted natriuretic peptide thresholds for a diagnosis of heart failure in the community: Diagnostic accuracy study

**DOI:** 10.1002/ehf2.15383

**Published:** 2025-07-27

**Authors:** Clare J. Taylor, Kathryn S. Taylor, Nicholas R. Jones, Jose M. Ordóñez‐Mena, Antoni Bayes‐Genis, F.D. Richard Hobbs

**Affiliations:** ^1^ Department of Applied Health Sciences, School of Health Sciences, College of Medicine and Health University of Birmingham Birmingham UK; ^2^ Nuffield Department of Primary Care Health Sciences University of Oxford Oxford UK; ^3^ Heart Institute University Hospital Germans Trias i Pujol Barcelona Spain

**Keywords:** diagnosis, diagnostic accuracy, heart failure, natriuretic peptide testing

## Abstract

**Background:**

European Society of Cardiology (ESC) chronic heart failure (HF) guidelines recommend a single N‐terminal pro‐B‐type natriuretic peptide (NT‐proBNP) threshold of ≥125 pg/mL for specialist referral in symptomatic patients; however, natriuretic peptide levels increase with age.

**Objectives:**

We aimed to assess NT‐proBNP test performance at age‐adjusted thresholds recently proposed by the ESC Heart Failure Association (HFA).

**Methods:**

Diagnostic accuracy study using linked primary and secondary care data (2004–2018) in England. NT‐proBNP test performance at ESC HFA age‐adjusted rule‐in thresholds (≥125 pg/mL, ≥250 pg/mL and ≥500 pg/mL for <50 years, 50–74 years and ≥75 years, respectively) and a high‐risk threshold (≥2000 pg/mL) was assessed overall, by sex and body mass index (BMI) with ESC's suggested threshold reductions for obesity.

**Results:**

Of 155 347 patients with NT‐proBNP tests performed, 14 585 (9.4%) were diagnosed with HF. Current ESC single threshold of ≥125 pg/mL had sensitivity 94.6% [95% confidence interval (CI) 94.2–95.0] and specificity 50.0% (49.7–50.3). Age‐adjusted thresholds had reduced sensitivity (83.5%, 88.5%, 84.4%) but increased specificity (77.6%, 67.8%, 63.5%) across the respective age groups. The high‐risk threshold had sensitivity 38.9% (38.1–39.7) and specificity 96.1% (96.0–96.2). A high BMI was associated with lower sensitivity at each age‐adjusted threshold, which improved with adjustment by obesity category. Test performance was similar in women and men.

**Conclusions:**

At ESC HFA age‐adjusted thresholds, the number of referrals required for HF diagnostic assessment are substantially reduced, but with some (likely lower risk) cases initially being undetected. Lower thresholds for patients with obesity are needed to avoid missing HF cases, but there is no need for adjustment by sex.

## Introduction

Early diagnosis of heart failure (HF) allows timely initiation of treatments which can prevent hospitalization, improve quality of life and extend survival.^1^ However, the key symptoms of HF—breathlessness, exhaustion and ankle swelling—are common and have a variety of causes.^2^, ^3^ Natriuretic peptides (NP) are an important part of the HF diagnostic pathway,^4^ and international guidelines recommend carrying out an NP test with referral for echocardiography and specialist assessment if the level is raised.^5^, ^6^, ^7^ A negative test can be useful to rule out HF and consider other causes, but this depends upon the threshold set.^4^ NP level at the time of diagnosis is also predictive of outcome including HF‐related hospitalization and survival.^8^

Echocardiography is a limited resource in most health systems globally; therefore, NP testing is important to prioritize those patients most likely to have HF for imaging and referral for specialist review.^9^ This also reduces the possibility of overdiagnosis. B‐type NP and N‐terminal pro‐B‐type NP tests are used in clinical practice, although NT‐proBNP is becoming the predominant test as it is more stable and also unaffected by neprilysin inhibition.^10^ The American College of Cardiology/American Heart Association/Heart Failure Society of America (ACC/AHA/HFSA)^6^ and European Society of Cardiology (ESC)^7^ HF guidelines currently recommend a single NT‐proBNP cut‐off below 125 pg/mL to rule out chronic HF in the community^7^; however, patient factors including age, sex and body mass index (BMI), as well as comorbidities and therapies, can influence the level found on testing.^11^

NT‐proBNP levels increase with age, which can be challenging for interpretation of test results in both older and younger patients.^12^ Older patients will be more likely to have a positive NT‐proBNP test requiring further investigation, and younger patients may be missed if the level is below the threshold value required for referral. Higher NT‐proBNP levels are also found in women compared with men.^13^ A high BMI is associated with lower NT‐proBNP levels, which could lead to patients with obesity being initially falsely reassured they do not have HF leading to diagnostic delay. Current guidelines have the same testing thresholds across age strata, sex, and BMI.

In 2023, the ESC Heart Failure Association (HFA) published a practical algorithm for early diagnosis of HF proposing age‐adjusted rule‐in NT‐proBNP thresholds: ≥125 pg/mL for patients aged under 50 years, ≥250 pg/mL for patients aged 50–74 years and ≥500 pg/mL for patients 75 years and over.^14^ The ESC HFA also suggested adjustment for obesity classes I, II and III by lowering the NT‐proBNP threshold by 25%, 30% and 40% in patients with BMI 30–34.9 kg/m^2^, 35–39.9 kg/m^2^ and ≥40 kg/m^2^, respectively.

Our aim was to assess NT‐proBNP test performance for HF diagnosis at the ESC HFA age‐adjusted thresholds overall, in women and men separately, and by BMI with additional adjustment by obesity category.

## Methods

We analysed the Diagnose‐NP diagnostic accuracy study dataset. The methods for the Diagnose‐NP study have been described elsewhere.^15^ In brief, it is a population‐based cohort using primary care data from the Clinical Practice Research Datalink (CPRD) Gold and Aurum databases linked to inpatient Hospital Episode Statistics (HES) admitted patient care data and Index of Multiple Deprivation socioeconomic data in England. The combined CPRD databases contain data from over 1400 general practices in the United Kingdom, which is around 15% of general practices overall, and have been shown to be representative of the general population.^16^

Patients aged 45 years and over in the two CPRD databases with an NT‐proBNP test in their primary care record between 1 January 2004 and 31 December 2018 were included. Patients entered the cohort on the date of their NT‐proBNP test and exited the cohort on the date of their HF diagnosis or 6 months after their NT‐proBNP test date if they were not diagnosed with HF. Patients with a previous diagnosis of HF were excluded. Patients were only included if their primary care records were deemed acceptable for research purposes (a CPRD quality measure), eligible for linkage and had been registered at a practice for at least 12 months. NT‐proBNP tests and HF diagnosis codes were identified in CPRD using clinical coding lists (see Supporting information S1) derived from the NHS terminology and classifications browser and the Quality and Outcomes Framework guidance.^17^

### NT‐proBNP testing (index test)

**Figure 1:**
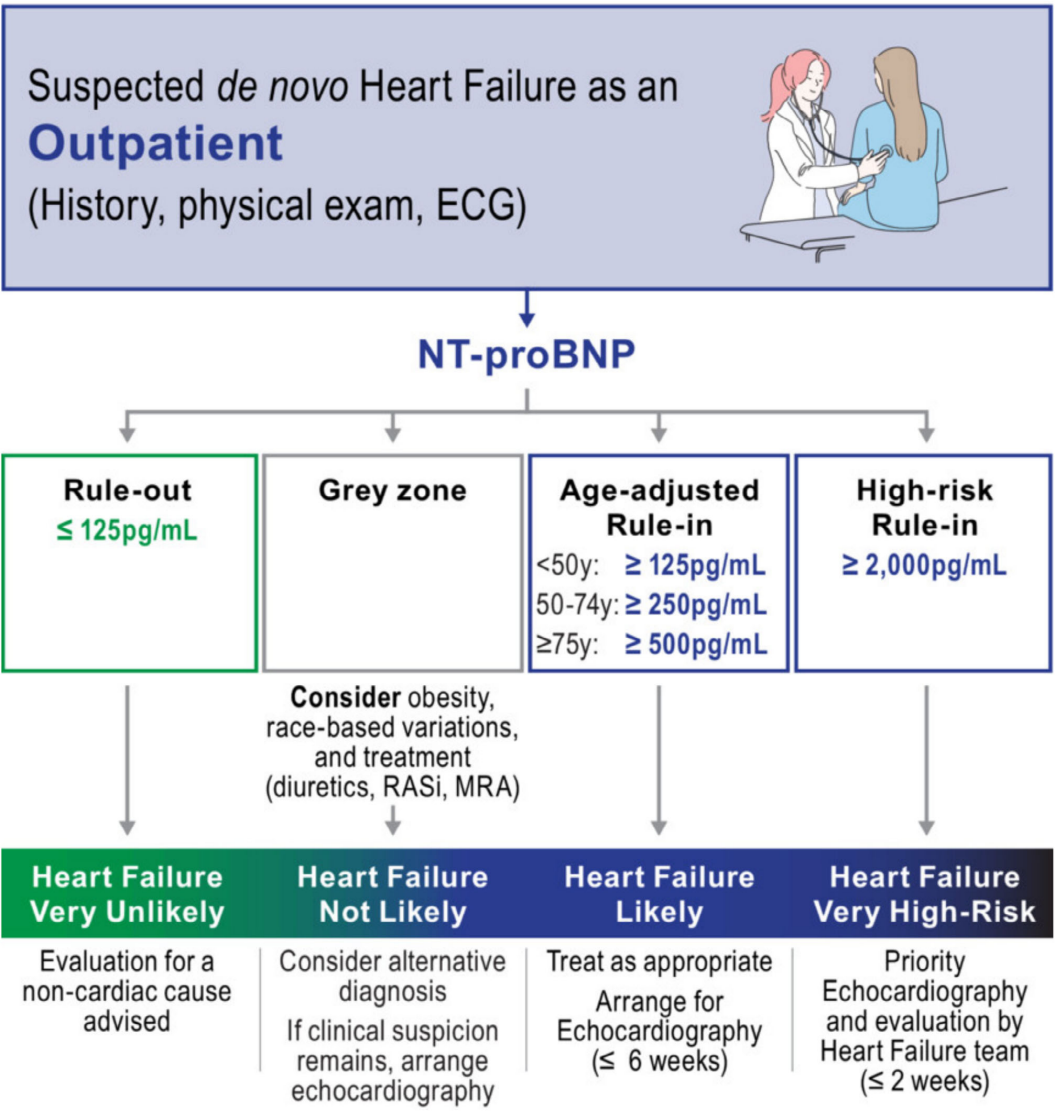
European Society of Cardiology Heart Failure Association algorithm for NT‐proBNP testing to diagnose heart failure in the outpatient setting. NT‐proBNP, N‐terminal pro‐B‐type natriuretic peptide.

NT‐proBNP level was analysed both as a continuous and categorical variable using ACC/AHA/HFSA and ESC chronic HF guideline (NT‐proBNP ≥125 pg/mL)^7^ and ESC HFA age‐adjusted^14^ (see *Figure* *1*) referral thresholds for chronic HF diagnosis. NT‐proBNP test performance at the ESC HFA high‐risk threshold (NT‐proBNP >2000 pg/mL) for rapid referral (to have echocardiography and specialist review within 2 weeks) was also explored. Subgroup analyses were carried out for women and men and by obesity category (including adjustment of the threshold for obesity categories I, II and III).

### HF diagnosis (reference standard)

The primary outcome of HF diagnosis within 6 months of the most recent NT‐proBNP test was obtained from either a diagnostic code entered in the CPRD database or from HES admitted patient care data based on hospital admission for HF or echocardiography findings consistent with HF. HF diagnoses from primary care were also validated through data linkage with HES using International Classification of Diseases, 10th revision codes (see Tables S1–S3).

### Statistical analysis

Sociodemographic variables were summarized with mean and standard deviation (*SD*) for continuous variables except NT‐proBNP, and frequencies and percentages for categorical variables. These were estimated for participants with a NT‐proBNP test overall, for participants split by age group (under 50 years, 50–74 years and ≥75 years), for women and men separately, and by BMI category (underweight <18.5 kg/m^2^, healthy weight 18.5–24.9 kg/m^2^, overweight 25–29.9 kg/m^2^, obesity class I 30.0–34.9 kg/m^2^, class II 35.0–39.9 kg/m^2^ and class III ≥ 40 kg/m^2^).

Diagnostic test accuracy for HF diagnosis was assessed by calculating sensitivity, specificity, positive predictive value (PPV), negative predictive value (NPV), likelihood ratio and diagnostic odds ratio using the ‘epitools’ package.^18^ Exact confidence intervals (CIs) for proportions were calculated using the binomial distribution. CIs for ratios were calculated using the Wald's normal approximation. Receiver operating characteristic (ROC) curves were plotted to assess overall test performance. The area under the ROC curve (AUC) was estimated using the ‘pROC’ package.^19^ All analyses were done in R (version 4.4.0) and used a 0.05 threshold to define statistical significance.

The protocol for the Diagnose‐NP study was approved by the Independent Scientific Advisory Committee (ISAC) of the Medicines and Healthcare products Regulatory Agency (MHRA) (ISAC protocol number 19_136). Ethics approval for observational research using CPRD with approval from ISAC was granted by a National Research Ethics Service committee (Trent MultiResearch Ethics Committee, reference number: 05/MRE04/87).

## Results

In total, 155 347 patients had an NT‐proBNP test recorded in their primary care record with a mean (*SD*) age of 61.1 years (11.0), more females (57.6%, *n* = 89 464) and the majority of White ethnicity (91.2%, *n* = 141 661). All five deprivation quintiles were equally represented (*Table* *1*). Of those with BMI recorded (96.3%), the mean BMI was 29.5 kg/m^2^ (6.4). Smoking was common with two thirds (67.2%) being current or former smokers. Long‐term conditions associated with developing HF were also prevalent in those undergoing testing, with hypertension (59.0%, *n* = 91 622) and diabetes (25.7%, *n* = 39 886) being particularly high. Compared with younger age‐groups, participants over 75 years were more likely to be female (64.9%, *n* = 10 841) and had more long‐term conditions except diabetes (21.0%, *n* = 3505). The proportion of those classed in the lowest deprivation quintile was highest in the under 50 years and lowest in those over 75 years (under 50 years 26.5%, *n* = 8722; 50–74 years 17.6%, *n* = 18 584; ≥75 years 13.3%, *n* = 2221). Smoking was more common in men compared with women (76.9% vs. 60.1%, respectively, being current or former smokers) (*Table* *1*). The deprivation quintiles and prevalence of long‐term conditions were similar across men and women. Compared with other BMI categories, those underweight were more likely to be female (75.1%, *n* = 1859), and those with obesity class III (BMI 30–34.9 kg/m^2^) were more likely to be in the lowest deprivation quintile (28.2%, *n* = 2773) and to have diabetes (43.2%, *n* = 4257) (*Table* *2*).

**Table 1:** Summary of characteristics for primary care patients undergoing NT‐proBNP testing overall and by age and sex.

Characteristic	All	<50 years	50–74 years	≥75 years	Men	Women
*N*	155 347	32 882	105 771	16 694	65 883	89 464
Age, years, mean (*SD*)	61.1 (11.0)	46.3 (1.7)	62.6 (6.7)	80.4 (4.4)	60.4 (10.77)	61.6 (11.3)
Sex, female *n* (%)	89 464 (57.6)	18 336 (55.8)	60 287 (57.0)	10 841 (64.9)	0 (0.0)	89 464 (100.0)
Ethnicity, *n* (%)						
White	141 661 (91.2)	28 266 (86.0)	97 722 (92.4)	15 673 (93.9)	60 290 (91.5)	81 371 (91.0)
Non‐White	10 946 (7.05)	3651 (11.1)	6451 (6.1)	844 (5.06)	4337 (6.58)	6609 (7.39)
Missing	2740 (1.8)	965 (2.9)	1598 (1.5)	177 (1.1)	1256 (1.9)	1484 (1.7)
BMI (kg/m^2^), mean (*SD*)	29.51 (6.42)	31.69 (7.39)	29.27 (6.06)	26.61 (5.01)	29.31 (5.75)	29.66 (6.87)
Smoking status, *n* (%)						
Never	50 629 (32.6)	10 859 (33.0)	33 002 (31.2)	6768 (40.5)	15 137 (23.0)	35 492 (39.7)
Former	79 714 (51.3)	14 240 (43.3)	57 059 (53.9)	8415 (50.4)	39 116 (59.4)	40 598 (45.4)
Current	24 691 (15.9)	7730 (23.5)	15 547 (14.7)	1414 (8.5)	11 497 (17.5)	13 194 (14.7)
Missing	313 (0.2)	53 (0.2)	163 (0.2)	97 (0.6)	133 (0.2)	180 (0.2)
IMD, quintile, *n* (%)						
Q1 (least deprived)	30 085 (19.4)	5202 (15.8)	21 193 (20.0)	3690 (22.1)	13 048 (19.8)	17 037 (19.0)
Q2	32 861 (21.2)	5748 (17.5)	23 288 (22.0)	3825 (22.9)	14 384 (21.8)	18 477 (20.7)
Q3	31 593 (20.3)	6098 (18.5)	21 768 (20.6)	3727 (22.3)	13 560 (20.6)	18 033 (20.2)
Q4	31 187 (20.1)	7086 (21.5)	20 879 (19.7)	3222 (19.3)	12 967 (19.7)	18 220 (20.4)
Q5 (most deprived)	29 527 (19.0)	8722 (26.5)	18 584 (17.6)	2221 (13.3)	11 884 (18.0)	17 643 (19.7)
Missing	94 (0.1)	26 (0.1)	59 (0.1)	9 (0.1)	40 (0.1)	54 (0.1)
Medical history, *n* (%)						
Diabetes	39 886 (25.7)	8077 (24.6)	28 304 (26.8)	3505 (21.0)	18 625 (28.3)	21 261 (23.8)
Hypertension	91 622 (59.0)	13 569 (41.3)	66 660 (63.0)	11 393 (68.2)	38 456 (58.4)	53 166 (59.4)
Atrial fibrillation	17 403 (11.2)	1162 (3.5)	13 210 (12.5)	3031 (18.2)	8960 (13.6)	8443 (9.4)
Angina	14 442 (9.3)	1413 (4.3)	10 941 (10.3)	2088 (12.5)	7663 (11.6)	6779 (7.6)
Ischaemic heart disease	18 007 (11.6)	2004 (6.1)	13 611 (12.9)	2392 (14.3)	10 443 (15.9)	7564 (8.5)
Myocardial infarction	9729 (6.3)	1256 (3.8)	7160 (6.8)	1313 (7.9)	6329 (9.6)	3400 (3.8)
Stroke	12 629 (8.1)	1269 (3.9)	9277 (8.8)	2083 (12.5)	6040 (9.2)	6589 (7.4)
Valvular disease	5689 (3.7)	506 (1.5)	4234 (4.0)	949 (5.7)	2410 (3.7)	3279 (3.7)
Other CV disease	21 438 (13.8)	2639 (8.0)	15 561 (14.7)	3238 (19.4)	9724 (14.8)	11 714 (13.1)
SBP (mmHg), mean (*SD*)	136.30 (16.90)	133.93 (16.65)	136.88 (16.74)	137.24 (17.95)	135.93 (16.49)	136.57 (17.19)
DBP (mmHg), mean (*SD*)	76.99 (10.32)	80.50 (10.21)	76.36 (10.06)	74.15 (10.43)	77.00 (10.53)	76.99 (10.16)
Total cholesterol (mmol/L), mean (*SD*)	4.87 (1.15)	5.10 (1.13)	4.81 (1.15)	4.79 (1.15)	4.53 (1.09)	5.12 (1.13)
NT‐pro BNP (pg/ml), median (IQR)	143 (60 413)	59 (30 123)	163 (74 448)	413 (174 1251)	134 (51 484)	148 (68 377)
Time between NP test and HF diagnosis (days)	26 (7.64)	24 (7.65)	27 (7.65)	22 (7.60)	27 (8.65)	24 (7.63)
<2 weeks, *n* (%)	5359 (36.7)	483 (38.3)	3722 (35.8)	1154 (39.6)	2606 (35.2)	2753 (38.3)
2–6 weeks, *n* (%)	3699 (25.4)	322 (25.6)	2641 (25.4)	736 (25.3)	1922 (26.0)	1777 (24.7)
≥6 weeks, *n* (%)	5527 (37.9)	455 (36.1)	4048 (38.9)	1024 (35.1)	2824 (38.8)	2653 (36.9)

Abbreviations: BMI, body mass index; BNP, B‐type natriuretic peptide; CV, cardiovascular; DBP, diastolic blood pressure; HF, heart failure; IMD, index of multiple deprivation; IQR, interquartile range (25th and 75th percentiles); NP, natriuretic peptide; Q, quintile; SBP, systolic blood pressure; *SD*, standard deviation.

**Table 2:** Summary of characteristics for primary care patients undergoing NT‐proBNP testing by BMI categories.

Characteristic	Underweight	Healthy	Overweight	Obesity I	Obesity II	Obesity III	Missing
*N*	2475	33 988	51 630	35 704	15 966	9843	5741
Age, years, mean (*SD*)	64.94 (11.6)	64.1 (11.3)	62.0 (10.7)	59.6 (10.2)	57.2 (9.7)	54.2 (8.9)	65.4 (13.7)
Sex, female *n* (%)	1859 (75.1)	20 672 (60.8)	26 938 (52.2)	19 997 (56.0)	9944 (62.3)	6719 (68.3)	3335 (58.1)
Ethnicity, *n* (%)							
White	2304 (93.1)	31 274 (92.0)	47 073 (91.2)	32 454 (90.9)	14 479 (90.7)	8955 (91.0)	5122 (89.2)
Non‐White	131 (5.29)	2136 (6.28)	3711 (7.19)	2706 (7.58)	1242 (7.78)	709 (7.2)	311 (5.42)
Missing	40 (1.6)	578 (1.7)	846 (1.6)	544 (1.5)	245 (1.5)	179 (1.8)	308 (5.4)
BMI (kg/m^2^), mean (*SD*)	17.08 (1.22)	22.67 (1.68)	27.49 (1.40)	32.21 (1.42)	37.13 (1.42)	44.74 (4.62)	—
Smoking status, *n* (%)							
Never	717 (29.0)	11 661 (34.3)	16 772 (32.5)	11 138 (31.2)	4830 (30.3)	2986 (30.3)	2525 (44.0)
Former	953 (38.5)	16 186 (47.6)	27 196 (52.7)	19 356 (54.2)	8636 (54.1)	5245 (53.3)	2142 (37.3)
Current	803 (32.4)	6117 (18.0)	7603 (14.7)	5172 (14.5)	2484 (15.6)	1610 (16.4)	902 (15.7)
Missing	2 (0.1)	24 (0.1)	59 (0.1)	38 (0.1)	16 (0.1)	2 (0.0)	172 (3.0)
IMD, quintile, *n* (%)							
Q1 (least deprived)	487 (19.7)	7525 (22.1)	10 893 (21.1)	6342 (17.8)	2430 (15.2)	1223 (12.4)	1185 (20.6)
Q2	445 (18.0)	7696 (22.6)	11 551 (22.4)	7220 (20.2)	2966 (18.6)	1661 (16.9)	1322 (23.0)
Q3	464 (18.7)	6896 (20.3)	10 432 (20.2)	7496 (21.0)	3185 (19.9)	1910 (19.4)	1210 (21.1)
Q4	486 (19.6)	6408 (18.9)	10 001 (19.4)	7352 (20.6)	3457 (21.7)	2269 (23.1)	1214 (21.1)
Q5 (most deprived)	591 (23.9)	5441 (16.0)	8726 (16.9)	7272 (20.4)	3916 (24.5)	2773 (28.2)	808 (14.1)
Missing	2 (0.1)	22 (0.1)	27 (0.1)	22 (0.1)	12 (0.1)	7 (0.1)	2 (0.0)
Medical history, *n* (%)							
Diabetes	366 (14.8)	5940 (17.5)	11 773 (22.8)	11 166 (31.3)	5896 (36.9)	4257 (43.2)	488 (8.5)
Hypertension	1103 (44.6)	17 570 (51.7)	30 196 (58.5)	22 913 (64.2)	10 815 (67.7)	6781 (68.9)	2244 (39.1)
Atrial fibrillation	260 (10.5)	4349 (12.8)	6059 (11.7)	3716 (10.4)	1523 (9.5)	861 (8.7)	635 (11.1)
Angina	167 (6.7)	3035 (8.9)	5255 (10.2)	3638 (10.2)	1369 (8.6)	727 (7.4)	251 (4.4)
Ischaemic heart disease	208 (8.4)	3876 (11.4)	6655 (12.9)	4437 (12.4)	1644 (10.3)	860 (8.7)	327 (5.7)
Myocardial infarction	124 (5.0)	2158 (6.3)	3668 (7.1)	2278 (6.4)	855 (5.4)	433 (4.4)	213 (3.7)
Stroke	227 (9.2)	3092 (9.1)	4514 (8.7)	2739 (7.7)	1084 (6.8)	549 (5.6)	424 (7.4)
Valvular disease	99 (4.0)	1581 (4.7)	2082 (4.0)	1142 (3.2)	406 (2.5)	199 (2.0)	180 (3.1)
Other CV disease	364 (14.7)	5220 (15.4)	7602 (14.7)	4718 (13.2)	1865 (11.7)	1043 (10.6)	626 (10.9)
SBP (mmHg), mean (*SD*)	131.50 (19.07)	134.75 (17.77)	136.21 (16.60)	137.06 (16.27)	137.45 (16.07)	138.08 (16.48)	137.26 (18.85)
DBP (mmHg), mean (*SD*)	73.52 (11.10)	75.20 (10.30)	76.59 (10.09)	77.78 (10.10)	78.77 (10.12)	79.85 (10.43)	78.11 (11.15)
Total cholesterol (mmol/L), mean (*SD*)	4.97 (1.13)	4.94 (1.15)	4.88 (1.16)	4.84 (1.15)	4.79 (1.14)	4.75 (1.10)	5.07 (1.14)
NT‐pro BNP (pg/mL), median (IQR)	305 (132 906)	204 (85 634)	145 (61 421)	119 (51 314)	104 (49 265)	101 (47 245)	184 (71 635)
Time between NP test and HF diagnosis (days)	22 (6.57)	24 (7.60)	26 (8.65)	28 (8.70)	28 (8.67.2)	27 (7.65)	18 (6.55.5)
<2 weeks, *n* (%)	151 (42.5)	1456 (37.9)	1745 (36.4)	1023 (35.0)	418 (33.6)	273 (35.9)	293 (43.4)
2–6 weeks, *n* (%)	78 (22.0)	993 (25.9)	1206 (25.2)	740 (25.3)	321 (25.8)	196 (25.8)	165 (24.4)
≥6 weeks, *n* (%)	126 (35.5)	1390 (36.2)	1838 (38.4)	1159 (39.7)	505 (40.6)	292 (38.4)	217 (32.1)

Abbreviations: BMI, body mass index; CV, cardiovascular; DBP, diastolic blood pressure; HF, heart failure; IMD, index of multiple deprivation; IQR, interquartile range; NT‐proBNP, N‐terminal pro‐B‐type natriuretic peptide; SBP, systolic blood pressure; *SD*, standard deviation.

### HF diagnosis

A total of 14 585 (9.4%) were diagnosed with HF within 6 months of NT‐proBNP testing overall, including a higher proportion of those over 75 years compared with the younger age groups (3.8%, 9.8% and 17.5% in those under 50 years, 50–74 years and ≥75 years, respectively) and more men (11.2%) than women (8.2%). The proportion of people diagnosed with HF decreased across increasing BMI categories, with the highest proportion in those underweight (14.3%, 11.3%, 9.3% and around 8% in those underweight, healthy weight, overweight and obese, respectively).

The time between NT‐proBNP test and subsequent HF diagnosis was a median of 26 days (IQR 7–64); however, nearly 4 in 10 people waited 6 weeks or more (37.9%, *n* = 5527) to receive a diagnosis (*Table* *1*). Overall, 5359 patients (36.7%) had a HF diagnosis within 2 weeks, and this only increased slightly to 45% (*n* = 2549) for those with a NT‐proBNP test above 2000 pg/mL. For those above this threshold, participants under 50 years had the shortest delays, with median time between test and diagnosis of 12 days (IQR 3–34), 53% (*n* = 207) had a diagnosis within 2 weeks and 20% (*n* = 79) waited over 6 weeks. For other subgroups (based on age, sex and BMI), the median time to diagnosis was about 15 to 20 days. In the high‐risk category of NT‐proBNP above 2000 pg/mL, around 45% had a diagnosis within 2 weeks and around 30% waited over 6 weeks.

### NT‐proBNP testing

Of 155 347 with NT‐proBNP tests, 32 882 (21.2%) were under 50 years, 105 771 (68.1%) were 50–74 years and 16 694 (10.7%) were aged 75 years or over. There were 65 883 (42.4%) men, and 89 464 (57.6%) women. The median NT‐proBNP level was 143 pg/mL (IQR 60–413) overall, 59 pg/mL (30–123) in people under 50 years, 163 pg/mL (74–448) in those aged 50–74 years, 413 pg/mL (174–1251) in those ≥75 years, 148 pg/mL (68–377) in women and 134 pg/mL (51–484) in men (*Table* *1*). Median NT‐proBNP was 305 pg/mL (132–906) in those underweight, 204 pg/mL (85–634) in those with healthy weight, 145 pg/mL (61–421) in those overweight, 119 pg/mL (51–134) with obesity class I, 104 pg/mL (49–265) with class II and 101 pg/mL (47–245) for those in class III (*Table* *2*).

The corresponding median NT‐proBNP levels in patients with a HF diagnosis were 1358 pg/mL (534–3230) overall, 831 pg/mL (206–2541) in those aged <50 years, 1301 pg/mL (529–3057) in those aged 50–74 years, 1866 pg/mL (741–4089) in participants aged 75 years and over, 1506 pg/mL (584–3471) in men, 1224 pg/mL (495–2989) in women, 1807 pg/mL (738–4699) in those underweight, 1881 pg/mL (703–4310) with those at healthy weight, 1412 pg/mL (560–3390) in those overweight, 1132 pg/mL (456–2528) for those with obesity class I, 868 pg/mL (371–1905) with class II and 792 pg/mL (274–1906) for those in class III.

### NT‐proBNP diagnostic test accuracy

For all those with an NT‐proBNP test, the current single NT‐proBNP threshold ≥125 pg/mL had sensitivity 94.6% (95% CI 94.2–95.0) and specificity 50.0% (49.7–50.3), and for high risk (≥2000 pg/mL) sensitivity 38.9% (38.1–39.7) and specificity 96.1% (96.0–96.2) (*Table* *3*).

**Table 3:** Diagnostic test accuracy parameters for algorithm for the diagnosis of de novo chronic heart failure using NT‐proBNP level, overall and for age subgroups, at the current ESC threshold and at thresholds adjusting for age and high risk.

Test	Rule‐in ≥125 pg/mL	Rule‐in age <50 years ≥125 pg/mL	Rule‐in age 50–74 years ≥250 pg/mL	Rule‐in age ≥75 years ≥500 pg/mL	Rule‐in age‐adjusted	Rule‐in high risk ≥2000 pg/mL
*N*	155 347	32 882	105 771	16 694	155 347	155 347
Prevalence, % (95% CI)	9.4 (9.2, 9.5)	3.8 (3.6, 4)	9.8 (9.7, 10)	17.5 (16.9, 18)	9.4 (9.2, 9.5)	9.4 (9.2, 9.5)
TP, *n*	13 801	1052	9218	2458	12 728	5674
FN, *n*	784	208	1193	456	1857	8911
FP, *n*	703	7069	30 753	5035	42 857	5424
TN, *n*	70 397	24 553	64 607	8745	97 905	135 338
Sensitivity, % (95% CI)	94.6 (94.2, 95)	83.5 (81.3, 85.5)	88.5 (87.9, 89.1)	84.4 (83, 85.7)	87.3 (86.7, 87.8)	38.9 (38.1, 39.7)
Specificity, % (95% CI)	50 (49.7, 50.3)	77.6 (77.2, 78.1)	67.8 (67.5, 68)	63.5 (62.7, 64.3)	69.6 (69.3, 69.8)	96.1 (96, 96.2)
PPV, % (95% CI)	16.4 (16.1, 16.6)	13 (12.2, 13.7)	23.1 (22.6, 23.5)	32.8 (31.7, 33.9)	22.9 (22.5, 23.2)	51.1 (50.2, 52.1)
NPV, % (95% CI)	98.9 (98.8, 99)	99.2 (99, 99.3)	98.2 (98.1, 98.3)	95 (94.6, 95.5)	98.1 (98.1, 98.2)	93.8 (93.7, 93.9)
LR+ (95% CI)	1.89 (1.88, 1.91)	3.73 (3.62, 3.86)	2.75 (2.71, 2.78)	2.31 (2.25, 2.37)	2.87 (2.84, 2.9)	10.1 (9.77, 10.44)
LR− (95% CI)	0.11 (0.1, 0.12)	0.21 (0.19, 0.24)	0.17 (0.16, 0.18)	0.25 (0.23, 0.27)	0.18 (0.18, 0.19)	0.64 (0.63, 0.64)
DOR (95% CI)	17.61 (16.38, 18.94)	17.55 (15.13, 20.46)	16.23 (15.26, 17.26)	9.36 (8.43, 10.42)	15.66 (14.9, 16.46)	15.89 (15.22, 16.59)

Abbreviations: CI, confidence interval; DOR, diagnostic odds ratio; ESC, European Society of Cardiology; FN, false negatives; FP, false positives; LR, likelihood ratio; NPV, negative predictive value; NT‐proBNP, N‐terminal pro‐B‐type natriuretic peptide; PPV, positive predictive value; TN, true negatives; TP, true positives.

**Figure 2:**
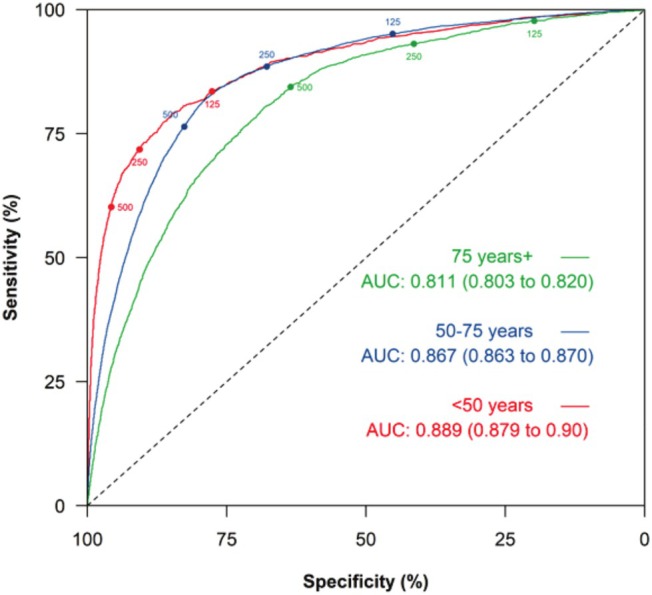
ROC curve for HF diagnosis for NT‐proBNP tests at ESC age‐specific referral thresholds of participants aged under 50 years, 50–74 years and ≥75 years. AUC, area under the ROC curve; ESC, European Society of Cardiology; HF, heart failure; NT‐proBNP, N‐terminal pro B‐type natriuretic peptide; ROC, receiver operating characteristic.

The ESC HFA age‐adjusted thresholds for <50 years, 50–74 years and ≥75 years had sensitivities of 83.5% (81.3–85.5), 88.5% (87.9–89.1) and 84.4% (83.0–85.7), respectively. The specificity was highest in the <50 years group at 77.6% (77.2–78.1), then 67.8% (67.5–68.0) in 50–74 years, and 63.5% (62.7–64.3) in ≥75 years, respectively (*Table* *4*). The overall performance in using NT‐proBNP was statistically significantly better for people under 50 years (AUC 0.89, 95% CI = 0.88 to 0.90) compared with other age groups (0.867, 95% CI 0.863–0.87 for age 50–74 years, 0.81, 95% CI 0.80–0.82 for age ≥75 years) (*Figure* *2*).

**Table 4:** Diagnostic test accuracy parameters for algorithm for the diagnosis of de novo chronic heart failure using NT‐proBNP level overall and for age subgroups, at the current ESC threshold and at thresholds adjusting for age and high risk, for women only.

Test	Rule‐in ≥125 pg/mL	Rule‐in age <50 years ≥125 pg/mL	Rule‐in age 50–74 years ≥250 pg/mL	Rule‐in age ≥75 years ≥500 pg/mL	Rule‐in age‐adjusted	Rule‐in high risk ≥2000 pg/mL
*N*	89 464	18 336	60 287	10 841	89 464	89 464
Prevalence, % (95% CI)	8 (7.9, 8.2)	2.8 (2.6, 3)	8.2 (8, 8.4)	15.9 (15.2, 16.6)	8 (7.9, 8.2)	8 (7.9, 8.2)
TP, *n*	6815	425	4350	1419	6194	2632
FN, *n*	368	86	603	300	989	4551
FP, *n*	43 139	4376	17 610	3227	25 213	2858
TN, *n*	39 142	13 449	37 724	5895	57 068	79 423
Sensitivity, % (95% CI)	94.9 (94.3, 95.4)	83.2 (79.6, 86.3)	87.8 (86.9, 88.7)	82.5 (80.7, 84.3)	86.2 (85.4, 87)	36.6 (35.5, 37.8)
Specificity, % (95% CI)	47.6 (47.2, 47.9)	75.5 (74.8, 76.1)	68.2 (67.8, 68.6)	64.6 (63.6, 65.6)	69.4 (69, 69.7)	96.5 (96.4, 96.7)
PPV, % (95% CI)	13.6 (13.3, 13.9)	8.9 (8.1, 9.7)	19.8 (19.3, 20.3)	30.5 (29.2, 31.9)	19.7 (19.3, 20.2)	47.9 (46.6, 49.3)
NPV, % (95% CI)	99.1 (99, 99.2)	99.4 (99.2, 99.5)	98.4 (98.3, 98.5)	95.2 (94.6, 95.7)	98.3 (98.2, 98.4)	94.6 (94.4, 94.7)
LR+ (95% CI)	1.81 (1.79, 1.82)	3.39 (3.23, 3.55)	2.76 (2.72, 2.8)	2.33 (2.25, 2.42)	2.81 (2.78, 2.85)	10.55 (10.06, 11.06)
LR− (95% CI)	0.11 (0.1, 0.12)	0.22 (0.18, 0.27)	0.18 (0.17, 0.19)	0.27 (0.24, 0.3)	0.2 (0.19, 0.21)	0.66 (0.64, 0.67)
DOR (95% CI)	16.8 (15.13, 18.71)	15.16 (12.06, 19.28)	15.45 (14.17, 16.87)	8.64 (7.58, 9.87)	14.17 (13.24, 15.19)	16.07 (15.12, 17.08)

Abbreviations: CI, confidence interval; DOR, diagnostic odds ratio; ESC, European Society of Cardiology; FN, false negatives; FP, false positives; LR, likelihood ratio; NPV, negative predictive value; NT‐proBNP, N‐terminal pro‐B‐type natriuretic peptide; PPV, positive predictive value; TN, true negatives; TP, true positives.

For NT‐proBNP testing, PPV improved across age groups from 13.0% (12.2–13.7), to 23.1% (22.6–23.5) and 32.8% (31.7–33.9) in <50 years, 50–74 years and ≥75 years groups, respectively, due to the increasing prevalence of HF with age. NPV was 95% or above across all categories (*Table* *3*).

**Figure 3:**
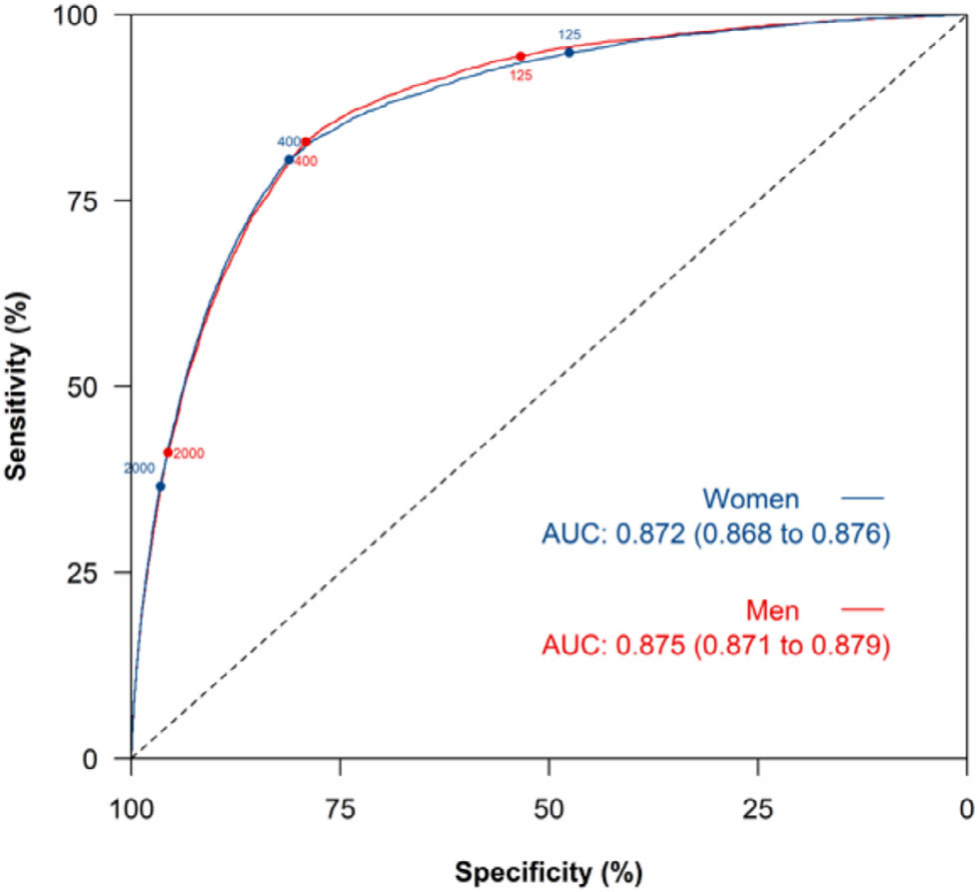
ROC curve for HF diagnosis for NT‐proBNP tests at ESC and NICE referral thresholds for men and women separately. AUC, area under the ROC curve; ESC, European Society of Cardiology; HF, heart failure; NICE, National Institute for Health and Care Excellence; NT‐proBNP, N‐terminal pro‐B‐type natriuretic peptide; ROC, receiver operating characteristic.

**Figure 4:**
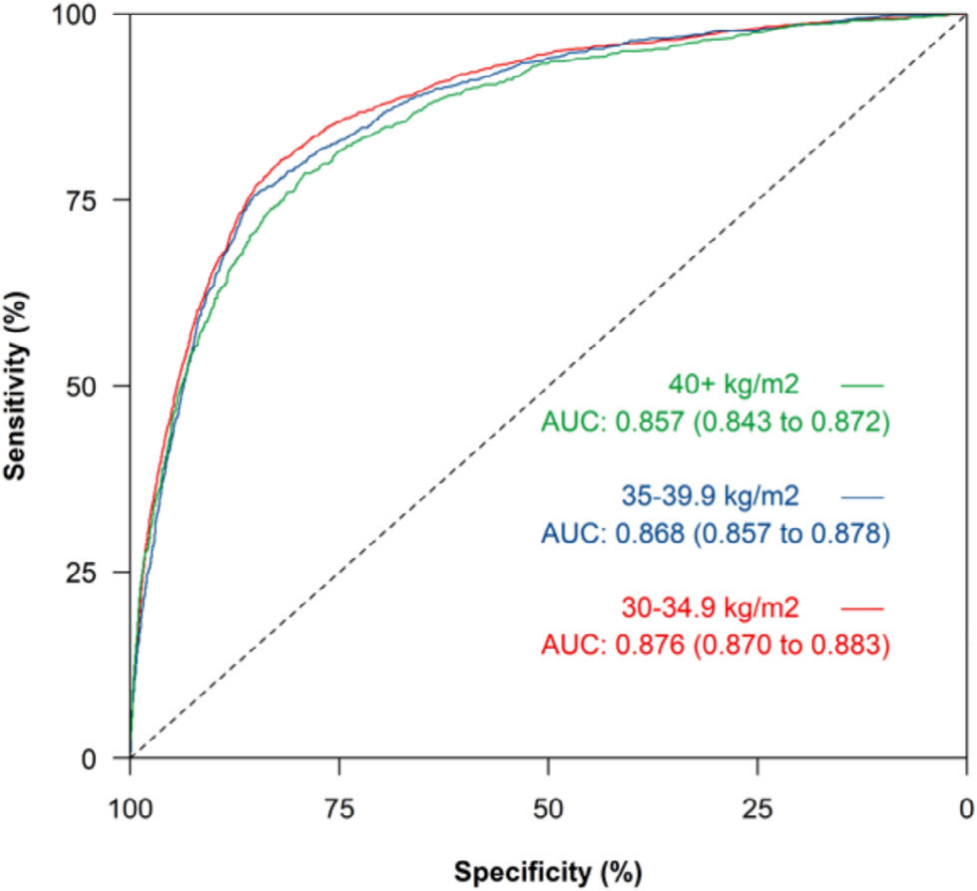
ROC curve for HF diagnosis for NT‐proBNP tests at ESC and NICE referral thresholds for obesity classes I, II and III. AUC, area under the ROC curve; ESC, European Society of Cardiology; HF, heart failure; NICE, National Institute for Health and Care Excellence; NT‐proBNP, N‐terminal pro B‐type natriuretic peptide; ROC, receiver operating characteristic.

Sensitivities at all three age groups were similar in women and men (*Tables* *4* and *5*, respectively). However, specificity was improved to above 80% for men below the age of 50 years. The overall performance of NT‐proBNP testing was similar for men and women (*Figure* *3*) and across the obesity classes (*Figure* *4*).

**Table 5:** Diagnostic test accuracy parameters for algorithm for the diagnosis of de novo chronic heart failure using NT‐proBNP level overall and for age subgroups, at the current ESC threshold and at thresholds adjusting for age and high risk, for men only.

Test	Rule‐in ≥125 pg/mL	Rule‐in age <50 years ≥125 pg/mL	Rule‐in age 50–74 years ≥250 pg/mL	Rule‐in age ≥75 years ≥500 pg/mL	Rule‐in age‐adjusted	Rule‐in high risk ≥2000 pg/mL
*N*	65 883	14 546	45 484	5853	65 883	65 883
Prevalence, % (95% CI)	11.2 (11, 11.5)	5.1 (4.8, 5.5)	12 (11.7, 12.3)	20.4 (19.4, 21.5)	11.2 (11, 11.5)	11.2 (11, 11.5)
TP, *n*	6986	627	4868	1039	6534	3042
FN, *n*	416	122	590	156	868	4360
FP, *n*	27 226	2693	13 143	1808	17 644	2566
TN, *n*	31 255	11 104	26 883	2850	40 837	55 915
Sensitivity, % (95% CI)	94.4 (93.8, 94.9)	83.7 (80.9, 86.3)	89.2 (88.3, 90)	86.9 (84.9, 88.8)	88.3 (87.5, 89)	41.1 (40, 42.2)
Specificity, % (95% CI)	53.4 (53, 53.8)	80.5 (79.8, 81.1)	67.2 (66.7, 67.6)	61.2 (59.8, 62.6)	69.8 (69.5, 70.2)	95.6 (95.4, 95.8)
PPV, % (95% CI)	20.4 (20, 20.9)	18.9 (17.6, 20.3)	27 (26.4, 27.7)	36.5 (34.7, 38.3)	27 (26.5, 27.6)	54.2 (52.9, 55.6)
NPV, % (95% CI)	98.7 (98.6, 98.8)	98.9 (98.7, 99.1)	97.9 (97.7, 98)	94.8 (94, 95.6)	97.9 (97.8, 98.1)	92.8 (92.6, 93)
LR+ (95% CI)	2.03 (2.01, 2.05)	4.29 (4.09, 4.49)	2.72 (2.67, 2.76)	2.24 (2.15, 2.34)	2.93 (2.88, 2.97)	9.37 (8.94, 9.81)
LR− (95% CI)	0.11 (0.1, 0.12)	0.2 (0.17, 0.24)	0.16 (0.15, 0.17)	0.21 (0.18, 0.25)	0.17 (0.16, 0.18)	0.62 (0.6, 0.63)
DOR (95% CI)	19.27 (17.46, 21.34)	21.16 (17.42, 25.93)	16.87 (15.46, 18.44)	10.49 (8.8, 12.57)	17.42 (16.2, 18.74)	15.2 (14.3, 16.15)

Abbreviations: CI, confidence interval; DOR, diagnostic odds ratio; ESC, European Society of Cardiology; FN, false negatives; FP, false positives; LR, likelihood ratio; NPV, negative predictive value; NT‐proBNP, N‐terminal pro‐B‐type natriuretic peptide; PPV, positive predictive value; TN, true negatives; TP, true positives.

For all age‐adjusted thresholds, sensitivity decreased with increasing obesity category (*Tables* *6*, *7*, *8*). By lowering the NT‐proBNP age‐adjusted thresholds by 25%, 30% and 40% in obesity category I, II and III, respectively, the sensitivity and specificity was comparable with the test performance seen in the cohort overall. The same comparable performance applied to those overweight and at healthy weight at the age‐adjusted thresholds (*Table*s *9* and *10*), but this did not apply to those who were underweight, for which specificity was below 30% at the ACC/AHA/HFSA and ESC 125 pg/mL threshold, and all specificities were consistently lower than those for the cohort overall (*Table* *11*).

**Table 6:** Diagnostic test accuracy parameters for algorithm for the diagnosis of de novo heart failure using NT‐proBNP level, at the current ESC threshold and at thresholds adjusting for age and high risk and reduced by 25% for people with obesity class I only.

Test	Rule‐in ≥125 pg/mL	Rule‐in ≥93.75 pg/mL	Rule‐in age <50 years ≥125 pg/mL	Rule‐in age <50 years ≥93.75 pg/mL	Rule‐in age 50–75 years ≥250 pg/mL	Rule‐in age 50–75 years ≥187.5 pg/mL	Rule‐in age 75 years+ ≥500 pg/mL	Rule‐in age 75 years+ ≥375 pg/mL	Rule‐in ≥2000 pg/mL	Rule‐in ≥1500 pg/mL
*N*	35 704	35 704	8205	8205	24 978	24 978	2521	2521	35 704	35 704
Prevalence, % (95% CI)	8.2 (7.9–8.5)	8.2 (7.9–8.5)	3.5 (3.1–3.9)	3.5 (3.1–3.9)	9 (8.6–9.3)	9 (8.6–9.3)	15.5 (14.1–17)	15.5 (14.1–17)	8.2 (7.9–8.5)	8.2 (7.9–8.5)
TP, *n*	2722	2784	238	246	1934	2004	312	335	955	1205
FN, *n*	200	138	49	41	310	240	79	56	1967	1717
FP, *n*	14 596	17 656	1601	2237	6407	8202	646	808	798	1265
TN, *n*	18 186	15 126	6317	5681	16 327	14 532	1484	1322	31 984	31 517
Sensitivity, % (95% CI)	93.2 (92.2–94)	95.3 (94.4–96)	82.9 (78.1–87.1)	85.7 (81.1–89.5)	86.2 (84.7–87.6)	89.3 (88–90.6)	79.8 (75.5–83.7)	85.7 (81.8–89)	32.7 (31–34.4)	41.2 (39.4–43)
Specificity, % (95% CI)	55.5 (54.9–56)	46.1 (45.6–46.7)	79.8 (78.9–80.7)	71.7 (70.7–72.7)	71.8 (71.2–72.4)	63.9 (63.3–64.5)	69.7 (67.7–71.6)	62.1 (60–64.1)	97.6 (97.4–97.7)	96.1 (95.9–96.3)
PPV, % (95% CI)	15.7 (15.2–16.3)	13.6 (13.2–14.1)	12.9 (11.4–14.6)	9.9 (8.8–11.2)	23.2 (22.3–24.1)	19.6 (18.9–20.4)	32.6 (29.6–35.6)	29.3 (26.7–32)	54.5 (52.1–56.8)	48.8 (46.8–50.8)
NPV, % (95% CI)	98.9 (98.8–99.1)	99.1 (98.9–99.2)	99.2 (99–99.4)	99.3 (99–99.5)	98.1 (97.9–98.3)	98.4 (98.2–98.6)	94.9 (93.7–96)	95.9 (94.8–96.9)	94.2 (94–94.5)	94.8 (94.6–95.1)
LR+ (95% CI)	2.09 (2.06–2.13)	1.77 (1.75–1.79)	4.1 (3.83–4.39)	3.03 (2.86–3.22)	3.06 (2.98–3.14)	2.48 (2.42–2.53)	2.63 (2.43–2.85)	2.26 (2.11–2.42)	13.43 (12.32–14.63)	10.69 (9.97–11.45)
LR− (95% CI)	0.12 (0.11–0.14)	0.1 (0.09–0.12)	0.21 (0.17–0.28)	0.2 (0.15–0.26)	0.19 (0.17–0.21)	0.17 (0.15–0.19)	0.29 (0.24–0.35)	0.23 (0.18–0.29)	0.69 (0.67–0.71)	0.61 (0.59–0.63)
DOR (95% CI)	16.94 (14.69–19.65)	17.26 (14.59–20.6)	19.11 (14.11–26.39)	15.18 (11–21.52)	15.89 (14.07–18.01)	14.78 (12.92–16.98)	9.05 (6.99–11.85)	9.76 (7.31–13.25)	19.45 (17.53–21.6)	17.48 (15.94–19.18)

Abbreviations: CI, confidence interval; DOR, diagnostic odds ratio; ESC, European Society of Cardiology; FN, false negatives; FP, false positives; LR, likelihood ratio; NPV, negative predictive value; NT‐proBNP, N‐terminal pro‐B‐type natriuretic peptide; PPV, positive predictive value; TN, true negatives; TP, true positives.

**Table 7:** Diagnostic test accuracy parameters for algorithm for the diagnosis of de novo heart failure using NT‐proBNP level, at the current ESC threshold and at thresholds adjusting for age and high risk and reduced by 40%, for people with obesity class II.

Test	Rule‐in ≥125 pg/mL	Rule‐in ≥87.5 pg/mL	Rule‐in age <50 years ≥125 pg/mL	Rule‐in age <50 years ≥87.5 pg/mL	Rule‐in age 50–75 years ≥250 pg/mL	Rule‐in age 50–75 years ≥175 pg/mL	Rule‐in age 75 years ≥500 pg/mL	Rule‐in age 75 years ≥350 pg/mL	Rule‐in ≥2000 pg/mL	Rule‐in ≥1400 pg/mL
*N*	15 966	15 966	4814	4814	10 490	10 490	662	662	15 966	15 966
Prevalence, % (95% CI)	7.8 (7.4–8.2)	7.8 (7.4–8.2)	4 (3.5–4.6)	4 (3.5–4.6)	9.2 (8.7–9.8)	9.2 (8.7–9.8)	12.8 (10.4–15.6)	12.8 (10.4–15.6)	7.8 (7.4–8.2)	7.8 (7.4–8.2)
TP, *n*	1133	1179	157	173	803	855	69	74	290	422
FN, *n*	111	65	35	19	164	112	16	11	954	822
FP, *n*	5966	7724	978	1446	2518	3485	150	210	284	506
TN, *n*	8756	6998	3644	3176	7005	6038	427	367	14 438	14 216
Sensitivity, % (95% CI)	91.1 (89.4–92.6)	94.8 (93.4–95.9)	81.8 (75.6–87)	90.1 (85–93.9)	83 (80.5–85.4)	88.4 (86.2–90.4)	81.2 (71.2–88.8)	87.1 (78–93.4)	23.3 (21–25.8)	33.9 (31.3–36.6)
Specificity, % (95% CI)	59.5 (58.7–60.3)	47.5 (46.7–48.3)	78.8 (77.6–80)	68.7 (67.4–70.1)	73.6 (72.7–74.4)	63.4 (62.4–64.4)	74 (70.2–77.5)	63.6 (59.5–67.5)	98.1 (97.8–98.3)	96.6 (96.3–96.9)
PPV, % (95% CI)	16 (15.1–16.8)	13.2 (12.5–14)	13.8 (11.9–16)	10.7 (9.2–12.3)	24.2 (22.7–25.7)	19.7 (18.5–20.9)	31.5 (25.4–38.1)	26.1 (21–31.6)	50.5 (46.4–54.7)	45.5 (42.2–48.7)
NPV, % (95% CI)	98.7 (98.5–99)	99.1 (98.8–99.3)	99 (98.7–99.3)	99.4 (99.1–99.6)	97.7 (97.3–98)	98.2 (97.8–98.5)	96.4 (94.2–97.9)	97.1 (94.9–98.5)	93.8 (93.4–94.2)	94.5 (94.2–94.9)
LR+ (95% CI)	2.25 (2.19–2.31)	1.81 (1.77–1.84)	3.86 (3.54–4.22)	2.88 (2.7–3.07)	3.14 (3.01–3.28)	2.42 (2.33–2.5)	3.12 (2.63–3.71)	2.39 (2.09–2.74)	12.08 (10.37–14.08)	9.87 (8.79–11.08)
LR− (95% CI)	0.15 (0.13–0.18)	0.11 (0.09–0.14)	0.23 (0.17–0.31)	0.14 (0.09–0.22)	0.23 (0.2–0.27)	0.18 (0.15–0.22)	0.25 (0.16–0.4)	0.2 (0.12–0.35)	0.78 (0.76–0.81)	0.68 (0.66–0.71)
DOR (95% CI)	14.96 (12.33–18.33)	16.39 (12.85–21.3)	16.64 (11.6–24.55)	19.84 (12.63–33.08)	13.61 (11.47–16.25)	13.21 (10.85–16.24)	12.14 (6.99–22.32)	11.58 (6.25–23.63)	15.45 (12.95–18.43)	14.42 (12.44–16.7)

Abbreviations: CI, confidence interval; DOR, diagnostic odds ratio; ESC, European Society of Cardiology; FN, false negatives; FP, false positives; LR, likelihood ratio; NPV, negative predictive value; NT‐proBNP, N‐terminal pro‐B‐type natriuretic peptide; PPV, positive predictive value; TN, true negatives; TP, true positives.

**Table 8:** Diagnostic test accuracy parameters for algorithm for the diagnosis of de novo heart failure using NT‐proBNP level, at the current ESC threshold and at thresholds adjusting for age and high risk and reduced by 50%, for people with obesity class III.

Test	Rule‐in ≥125 pg/mL	Rule‐in ≥75 pg/mL	Rule‐in age <50 years ≥125 pg/mL	Rule‐in age <50 years ≥75 pg/mL	Rule‐in age 50 to 74 years ≥250 pg/mL	Rule‐in age 50 to 74 years ≥150 pg/mL	Rule‐in age 75 years+ ≥500 pg/mL	Rule‐in age 75 years+ ≥300 pg/mL	Rule‐in ≥2000 pg/mL	Rule‐in ≥1200 pg/mL
*N*	9843	9843	4120	4120	5513	5513	210	210	9843	9843
Prevalence, % (95% CI)	7.7 (7.2–8.3)	7.7 (7.2–8.3)	5 (4.3–5.7)	5 (4.3–5.7)	9.5 (8.7–10.3)	9.5 (8.7–10.3)	15.2 (10.7–20.8)	15.2 (10.7–20.8)	7.7 (7.2–8.3)	7.7 (7.2–8.3)
TP, *n*	679	718	163	182	428	469	21	23	183	280
FN, *n*	82	43	42	23	96	55	11	9	578	481
FP, *n*	3531	5157	993	1700	1311	2085	44	71	123	333
TN, *n*	5551	3925	2922	2215	3678	2904	134	107	8959	8749
Sensitivity, % (95% CI)	89.2 (86.8–91.3)	94.3 (92.5–95.9)	79.5 (73.3–84.8)	88.8 (83.6–92.8)	81.7 (78.1–84.9)	89.5 (86.6–92)	65.6 (46.8–81.4)	71.9 (53.3–86.3)	24 (21.1–27.2)	36.8 (33.4–40.3)
Specificity, % (95% CI)	61.1 (60.1–62.1)	43.2 (42.2–44.2)	74.6 (73.2–76)	56.6 (55–58.1)	73.7 (72.5–74.9)	58.2 (56.8–59.6)	75.3 (68.3–81.4)	60.1 (52.5–67.4)	98.6 (98.4–98.9)	96.3 (95.9–96.7)
PPV, % (95% CI)	16.1 (15–17.3)	12.2 (11.4–13.1)	14.1 (12.1–16.2)	9.7 (8.4–11.1)	24.6 (22.6–26.7)	18.4 (16.9–19.9)	32.3 (21.2–45.1)	24.5 (16.2–34.4)	59.8 (54.1–65.3)	45.7 (41.7–49.7)
NPV, % (95% CI)	98.5 (98.2–98.8)	98.9 (98.5–99.2)	98.6 (98.1–99)	99 (98.5–99.3)	97.5 (96.9–97.9)	98.1 (97.6–98.6)	92.4 (86.8–96.2)	92.2 (85.8–96.4)	93.9 (93.4–94.4)	94.8 (94.3–95.2)
LR+ (95% CI)	2.29 (2.21–2.38)	1.66 (1.62–1.7)	3.13 (2.87–3.42)	2.04 (1.92–2.17)	3.11 (2.92–3.31)	2.14 (2.05–2.24)	2.65 (1.85–3.8)	1.8 (1.36–2.39)	17.76 (14.3–22.04)	10.03 (8.72–11.55)
LR− (95% CI)	0.18 (0.14–0.22)	0.13 (0.1–0.17)	0.27 (0.21–0.36)	0.2 (0.13–0.29)	0.25 (0.21–0.3)	0.18 (0.14–0.23)	0.46 (0.28–0.74)	0.47 (0.27–0.82)	0.77 (0.74–0.8)	0.66 (0.62–0.69)
DOR (95% CI)	12.99 (10.36–16.52)	12.66 (9.4–17.53)	11.38 (8.12–16.3)	10.24 (6.75–16.31)	12.49 (9.96–15.8)	11.84 (8.99–15.91)	5.72 (2.59–13.31)	3.79 (1.7–9.17)	23.03 (18.08–29.44)	15.28 (12.72–18.37)

Abbreviations: CI, confidence interval; DOR, diagnostic odds ratio; ESC, European Society of Cardiology; FN, false negatives; FP, false positives; LR, likelihood ratio; NPV, negative predictive value; NT‐proBNP, N‐terminal pro‐B‐type natriuretic peptide; PPV, positive predictive value; TN, true negatives; TP, true positives.

**Table 9:** Diagnostic test accuracy parameters for algorithm for the diagnosis of de novo heart failure using NT‐proBNP level, at the current ESC threshold and at thresholds adjusting for age and high risk, for people who are at overweight.

Test	Rule‐in ≥125 pg/mL	Rule‐in age <50 years ≥125 pg/mL	Rule‐in age 50–74 years ≥250 pg/mL	Rule‐in age ≥75 years ≥500 pg/mL	Rule‐in age‐adjusted	Rule‐in high risk ≥2000 pg/mL
*N*	51 630	9164	36 773	5693	51 630	51 630
Prevalence, % (95% CI)	9.3 (9, 9.5)	3.3 (3, 3.7)	9.7 (9.4, 10)	16.3 (15.3, 17.3)	9.3 (9, 9.5)	9.3 (9, 9.5)
TP, *n*	4537	250	3173	772	4195	1905
FN, *n*	252	54	384	156	594	2884
FP, *n*	23 792	1847	10 598	1648	14 093	1836
TN, *n*	23 049	7013	22 618	3117	32 748	45 005
Sensitivity, % (95% CI)	94.7 (94.1, 95.4)	82.2 (77.5, 86.4)	89.2 (88.1, 90.2)	83.2 (80.6, 85.5)	87.6 (86.6, 88.5)	39.8 (38.4, 41.2)
Specificity, % (95% CI)	49.2 (48.8, 49.7)	79.2 (78.3, 80)	68.1 (67.6, 68.6)	65.4 (64, 66.8)	69.9 (69.5, 70.3)	96.1 (95.9, 96.3)
PPV, % (95% CI)	16 (15.6, 16.4)	11.9 (10.6, 13.4)	23 (22.3, 23.8)	31.9 (30, 33.8)	22.9 (22.3, 23.6)	50.9 (49.3, 52.5)
NPV, % (95% CI)	98.9 (98.8, 99)	99.2 (99, 99.4)	98.3 (98.2, 98.5)	95.2 (94.4, 95.9)	98.2 (98.1, 98.4)	94 (93.8, 94.2)
LR+ (95% CI)	1.87 (1.84, 1.89)	3.94 (3.69, 4.21)	2.8 (2.74, 2.85)	2.41 (2.29, 2.53)	2.91 (2.86, 2.96)	10.15 (9.59, 10.74)
LR− (95% CI)	0.11 (0.09, 0.12)	0.22 (0.18, 0.29)	0.16 (0.14, 0.17)	0.26 (0.22, 0.3)	0.18 (0.16, 0.19)	0.63 (0.61, 0.64)
DOR (95% CI)	17.43 (15.37, 19.87)	17.53 (13.11, 23.85)	17.63 (15.84, 19.66)	9.35 (7.81, 11.25)	16.41 (15.03, 17.93)	16.19 (15.03, 17.44)

Abbreviations: CI, confidence interval; DOR, diagnostic odds ratio; ESC, European Society of Cardiology; FN, false negatives; FP, false positives; LR, likelihood ratio; NPV, negative predictive value; NT‐proBNP, N‐terminal pro‐B‐type natriuretic peptide; TN, true negatives; PPV, positive predictive value; TP, true positives.

**Table 10:** Diagnostic test accuracy parameters for algorithm for the diagnosis of de novo heart failure using NT‐proBNP level, at the current ESC threshold and at thresholds adjusting for age and high risk, for people who are at a healthy weight.

Test	Rule‐in ≥125 pg/mL	Rule‐in age <50 years ≥125 pg/mL	Rule‐in age 50–74 years ≥250 pg/mL	Rule‐in age ≥75 years ≥500 pg/mL	Rule‐in age‐adjusted	Rule‐in high risk ≥2000 pg/mL
*N*	33 988	5146	23 217	5625	33 988	33 988
Prevalence, % (95% CI)	11.3 (11, 11.6)	3.6 (3.1, 4.1)	11 (10.6, 11.4)	19.4 (18.4, 20.5)	11.3 (11, 11.6)	11.3 (11, 11.6)
TP, *n*	3734	166	2367	951	3484	1855
FN, *n*	105	18	195	142	355	1984
FP, *n*	18 144	1263	8247	1875	11 385	1884
TN, *n*	12 005	3699	12 408	2657	18 764	28 265
Sensitivity, % (95% CI)	97.3 (96.7, 97.8)	90.2 (85, 94.1)	92.4 (91.3, 93.4)	87 (84.9, 88.9)	90.8 (89.8, 91.7)	48.3 (46.7, 49.9)
Specificity, % (95% CI)	39.8 (39.3, 40.4)	74.5 (73.3, 75.8)	60.1 (59.4, 60.7)	58.6 (57.2, 60.1)	62.2 (61.7, 62.8)	93.8 (93.5, 94)
PPV, % (95% CI)	17.1 (16.6, 17.6)	11.6 (10, 13.4)	22.3 (21.5, 23.1)	33.7 (31.9, 35.4)	23.4 (22.8, 24.1)	49.6 (48, 51.2)
NPV, % (95% CI)	99.1 (99, 99.3)	99.5 (99.2, 99.7)	98.5 (98.2, 98.7)	94.9 (94, 95.7)	98.1 (97.9, 98.3)	93.4 (93.2, 93.7)
LR+ (95% CI)	1.62 (1.6, 1.63)	3.54 (3.31, 3.79)	2.31 (2.27, 2.36)	2.1 (2.02, 2.19)	2.4 (2.36, 2.45)	7.73 (7.32, 8.17)
LR− (95% CI)	0.07 (0.06, 0.08)	0.13 (0.08, 0.2)	0.13 (0.11, 0.15)	0.22 (0.19, 0.26)	0.15 (0.13, 0.16)	0.55 (0.53, 0.57)
DOR (95% CI)	23.49 (19.43, 28.74)	26.78 (16.86, 45.31)	18.25 (15.77, 21.25)	9.48 (7.9, 11.46)	16.17 (14.48, 18.11)	14.02 (12.97, 15.17)

Abbreviations: CI, confidence interval; DOR, diagnostic odds ratio; ESC, European Society of Cardiology; FN, false negatives; FP, false positives; LR, likelihood ratio; NPV, negative predictive value; NT‐proBNP, N‐terminal pro‐B‐type natriuretic peptide; PPV, positive predictive value; TN, true negatives; TP, true positives.

**Table 11:** Diagnostic test accuracy parameters for algorithm for the diagnosis of de novo heart failure using NT‐proBNP level, at the current ESC threshold and at thresholds adjusting for age and high risk, for people who are underweight.

Test	Rule‐in ≥125 pg/mL	Rule‐in age <50 years ≥125 pg/mL	Rule‐in age 50–74 years ≥250 pg/mL	Rule‐in age ≥75 years ≥500 pg/mL	Rule‐in age‐adjusted	Rule‐in high risk ≥2000 pg/mL
*N*	2475	346	1648	481	2475	2475
Prevalence, % (95% CI)	14.3 (13, 15.8)	7.5 (5, 10.8)	13.8 (12.2, 15.6)	21 (17.4, 24.9)	14.3 (13, 15.8)	14.3 (13, 15.8)
TP, *n*	344	20	218	95	333	169
FN, *n*	11	6	10	6	22	186
FP, *n*	1549	151	726	166	1043	168
TN, *n*	571	169	694	214	1077	1952
Sensitivity, % (95% CI)	96.9 (94.5, 98.4)	76.9 (56.4, 91)	95.6 (92.1, 97.9)	94.1 (87.5, 97.8)	93.8 (90.8, 96.1)	47.6 (42.3, 52.9)
Specificity, % (95% CI)	26.9 (25.1, 28.9)	52.8 (47.2, 58.4)	48.9 (46.2, 51.5)	56.3 (51.2, 61.4)	50.8 (48.7, 53)	92.1 (90.8, 93.2)
PPV, % (95% CI)	18.2 (16.5, 20)	11.7 (7.3, 17.5)	23.1 (20.4, 25.9)	36.4 (30.6, 42.6)	24.2 (22, 26.6)	50.1 (44.7, 55.6)
NPV, % (95% CI)	98.1 (96.6, 99.1)	96.6 (92.7, 98.7)	98.6 (97.4, 99.3)	97.3 (94.2, 99)	98 (97, 98.7)	91.3 (90, 92.5)
LR+ (95% CI)	1.33 (1.28, 1.37)	1.63 (1.28, 2.07)	1.87 (1.76, 1.98)	2.15 (1.9, 2.44)	1.91 (1.81, 2.01)	6.01 (5.01, 7.2)
LR− (95% CI)	0.12 (0.06, 0.21)	0.44 (0.21, 0.89)	0.09 (0.05, 0.16)	0.11 (0.05, 0.23)	0.12 (0.08, 0.18)	0.57 (0.51, 0.63)
DOR (95% CI)	11.36 (6.5, 22.24)	3.65 (1.5, 10.36)	20.5 (11.37, 41.81)	19.83 (9.18, 52.41)	15.51 (10.23, 24.82)	10.54 (8.12, 13.7)

Abbreviations: CI, confidence interval; DOR, diagnostic odds ratio; ESC, European Society of Cardiology; FN, false negatives; FP, false positives; LR, likelihood ratio; NPV, negative predictive value; NT‐proBNP, N‐terminal pro‐B‐type natriuretic peptide; PPV, positive predictive value; TN, true negatives; TP, true positives.

## Discussion

### Summary of findings

In this real‐world diagnostic accuracy study, ESC HFA age‐adjusted NT‐proBNP thresholds were associated with lower sensitivity but higher specificity compared with the single ACC/AHA/HFSA and ESC recommended threshold of 125 pg/mL. The performance at these thresholds was similar in women and men. Obesity categories were associated with lower sensitivity, which improved using the adjustment factors suggested by ESC HFA. A very elevated NT‐proBNP >2000 pg/mL was highly suggestive of HF.

### Strengths and limitations

This study used real‐world evidence to evaluate NT‐proBNP test accuracy in 155 347 primary care patients who underwent testing. This is a much larger sample size than any prospective HF diagnostic accuracy study in primary care to date.^20^ The large dataset allowed further exploration of age‐adjusted thresholds overall, in both sexes, and by obesity category, which would not be possible in smaller studies.

There are limitations of routinely collected data including accuracy of clinical coding. The reference standard was the presence or absence of a diagnostic code of HF, and the codes entered into primary care records are for clinical use rather than research purposes. However, HF as a condition is generally well recorded over the time the study was conducted.^21^ The type of HF—HF with reduced ejection fraction (HFrEF) or HF with preserved ejection fraction (HFpEF)—was also not possible to establish given the limited use of HFrEF and HFpEF codes in primary and secondary care records during the study period.^22^

The study was performed in England where the National Institute for Health and Care Excellence (NICE) recommend a NT‐proBNP threshold of 400 pg/mL for referral for outpatient diagnostic assessment.^5^ Patients with HF and a test result below the NICE threshold may therefore not have initially been referred via this diagnostic pathway so could potentially appear as false negative cases. However, these patients are likely to have presented to the healthcare system via other routes such as emergency admission, which is why a limit of 6 months was set to allow time for a HF diagnostic code to be entered in the primary care record.

The NP level can be influenced by factors we did not explore such as other long‐term conditions and medications.^22^ However, ACC/AHA/HFSA and ESC guidelines do not currently recommend stratifying by these factors, so our findings reflect current practice. Moreover, patients with long‐term conditions, which raise their NP level, particularly atrial fibrillation (AF) and chronic kidney disease (CKD), are likely to be captured in the higher age‐adjusted thresholds, so we focused on the impact of BMI in this analysis.

### Comparison with existing literature

Epidemiological and mechanistic studies have previously shown that NP levels increase with age.^23^, ^24^, ^25^, ^26^ In 911 healthy adults from the Framingham Heart Study in the United States, the strongest predictors of higher NP levels were older age and female sex.^23^ A population‐based cohort study of 2459 older adults in Germany also reported an age‐related incremental increase in NT‐proBNP levels. In apparently healthy individuals aged 65 years and over, 27% of men and 45% of women had a NT‐proBNP level above the 125 pg/mL threshold recommended by ACC/AHA/HFSA and ESC guidelines.^25^ The need for age adjusted thresholds to allow meaningful interpretation of NP results has therefore previously been proposed. A study of 5508 primary care patients, published in 2010, suggested thresholds of 50 pg/mL, 75 pg/mL and 250 pg/mL for age groups <50 years, 50–75 years and above 75 years, respectively, to improve test performance.^12^ However, these thresholds are yet to be adopted in international guidance. A recent paper describing the conceptual basis for a ‘Universal Definition of Heart Failure’ acknowledged the impact of age and sex on NP level and suggested potential diagnostic thresholds stratified by age and sex.^27^ In increments of 10 years from less than age 60 years to above age 80 years the threshold, starting at >75 pg/mL for men age less than 60 years, increased by 50 pg/mL for each decade and was 50 pg/mL higher for women than men in the same age group. These are much lower than the ESC HFA age‐adjusted thresholds tested in our study. The sensitivities at the lower thresholds are likely to be better, but many more echocardiography assessments would be needed, potentially overwhelming the service. Our data also suggest adjustment by sex is not necessary in contrast to previous studies.

The inverse relationship between BMI and NP levels leads to challenges in the interpretation of NT‐proBNP test results in patients with obesity and suspected HF. Potential mechanisms for lower NP levels in the presence of obesity include higher glomerular filtration, peri‐atrial fat reducing stretch and high expression of NT‐proBNP receptors on adipocytes reducing circulating levels.^28^ The relationship may be bidirectional whereby lack of circulating NP makes patients more prone to obesity, as well as obesity lowering NP levels. Once diagnosed, an obesity paradox has been observed where survival rates are better in people with overweight and obesity compared with normal weight,^29^ although NT‐proBNP level remains an independent predictor of prognosis and the obesity paradox has more recently been questioned.^8^, ^30^ Our findings suggest the need for NT‐proBNP age‐adjusted thresholds to avoid missing more cases of HF in patients living with obesity.

Conversely, specificity of NT‐proBNP was particularly low in the underweight group. There may be several clinical explanations for this. Patients with a low BMI may have an underlying condition, such as malignancy or frailty, which is associated with higher NT‐proBNP levels, so their test result was above the threshold for referral, but they did not have a diagnosis of HF.^31^

### Implications for policy and practice

In the United Kingdom, national guidance from NICE recommends that symptomatic patients are referred and have echocardiography and specialist assessment within 6 weeks if NT‐proBNP is 400 pg/mL or above, and within 2 weeks if NT‐proBNP is over 2000 pg/mL.^5^ However, healthcare systems globally have finite budgets and are increasingly under strain due to resource constraints. Patients are frequently hospitalized to receive a diagnosis of HF, and our UK data show most patients, including those at highest risk (NT‐proBNP >2000 pg/mL), wait more than the recommended time frames for assessment.^8^ NP testing needs to be an effective triage tool to ensure those patients at highest risk have rapid access to echocardiography and specialist assessment to allow treatment initiation in the community and prevent hospitalization. The use of age‐adjusted cut‐points could ensure older patients are not unnecessarily investigated for HF, avoiding the possibility of overdiagnosis, and younger and higher risk patients are seen sooner. This would mean more cases of HF may initially be missed in primary care, but with fewer people meeting the criteria for referral easing the burden on echocardiography and specialist cardiology services. For example, in our cohort, over a 14 year period, using the age‐adjusted thresholds in place of the ACC/AHA/HFSA and ESC 125 pg/mL threshold would result in 1073 missed HF cases (~77 per year) but saving 27 508 echocardiography and specialist assessment appointments (~2000 per year). Therefore, overall, the age‐adjusted thresholds would lead to an 18% reduction in unnecessary referrals, but with around 7.4% of all those with HF missed by the new approach. The cases that are initially undetected are likely to be low risk given our work showing baseline NT‐proBNP level is directly related to HF‐related hospitalization and mortality.^8^ Therefore, while some in the ‘Grey Zone’ between 125 pg/mL and the age‐adjusted thresholds could be diagnosed later this would free up capacity to allow those with high NT‐proBNP values (who are currently waiting too long) to undergo echocardiography and specialist assessment. The new algorithm could therefore mean those most likely to benefit can be seen and treated more quickly.

This trade‐off between a missed diagnosis and capacity within the HF diagnostic pathway is challenging for primary care clinicians, HF specialists and for patients and their families. Generalists need to be aware of all the factors that influence NP levels and take these into account when interpreting test results and making the decision to refer for echocardiography and specialist assessment. For patients with an NP result near to the referral threshold, clinical acumen and repeat NP testing may be useful for those where a HF diagnosis is suspected. The findings of this study should inform future guidelines to ensure recommended NP testing thresholds are evidence‐based and include adjustments for patient characteristics such as age and obesity. NT‐proBNP is currently still the most studied and reliable blood marker for HF diagnosis, but clinicians need to consider the impact of patient factors when interpreting test results and deciding, with the patient, on the most appropriate next step in the clinical pathway.

More research is needed to explore the practicalities of implementing age‐adjusted thresholds in practice, and further guidance is needed for how clinicians should manage patients in the ‘Grey Zone’. Further work is also needed to consider threshold recommendations in key subgroups, such as AF and CKD although these factors both increase NP levels, so are more likely to fall within the age‐adjusted rule in thresholds presented here. The ESC HFA position paper on age‐adjusted NT‐proBNP thresholds suggested the cut‐points should be reduced by 25%, 30% and 40% for class I, II and III obesity, respectively, although this recommendation was based on consensus.^14^ Our findings support the use of these adjustment factors by obesity category to avoid missing HF cases.

Globally, healthcare budgets and diagnostic pathways vary widely, and equitable allocation of healthcare resources for people with HF remains a challenge. The optimal NT‐proBNP threshold will ultimately depend on the priorities and capacity of the national healthcare system.

## Conflict of interest statement

C. T. reports consultancy fees from AstraZeneca, Roche, Bayer and Edwards outside the submitted work. A. B. G. reports lectures or advisory for Abbott, AskBio, AstraZeneca, Boehringer‐Ingelheim, Bayer, Medtronic, Novartis, Roche Diagnostics and Vifor.

## Funding

The original Diagnose‐NP study was funded by the National Institute for Health and Care Research Collaboration for Leadership in Applied Health Research and Care (CLAHRC) Oxford. The funders did not have any role in the design of the study, analysis and interpretation of the data or writing of the results for publication.

## Supporting information


**Table S1.** Clinical codes used to identify heart failure in CPRD.
**Table S2.** Clinical codes used to identify heart failure in hospital records.
**Table S3.** Clinical codes used to identify natriuretic peptide tests.
