# Supplementary material for: Age‐adjusted natriuretic peptide thresholds for a diagnosis of heart failure in the community: Diagnostic accuracy study
Source: ESC Heart Fail. 2025 Jul 27;12(5):3552–68. doi: 10.1002/ehf2.15383 (PMC12450762; doi:10.1002/ehf2.15383)
Supplement: Supplementary file 1 — Table S1. Clinical codes used to identify heart failure in CPRD. Table S2. Clinical codes used to identify heart failure in hospital records. Table S3. Clinical codes used to identify natriuretic peptide tests. [file EHF2-12-3552-s001.docx]

**Supplementary material**

**Tables**

**Table S1** Clinical codes used to identify heart failure in CPRD

| **Medcode** | **Clinical Code** | **Read Term** |
| --- | --- | --- |
| 398 | G580.00 | Congestive heart failure |
| 884 | G581.00 | Left ventricular failure |
| 2062 | G58..00 | Heart failure |
| 2906 | G580.11 | Congestive cardiac failure |
| 12627 | 9N0k.00 | Seen in heart failure clinic |
| 4024 | G58z.00 | Heart failure NOS |
| 19002 | 9N2p.00 | Seen by community heart failure nurse |
| 17851 | 8HBE.00 | Heart failure follow-up |
| 12366 | 662T.00 | Congestive heart failure monitoring |
| 30779 | 662W.00 | Heart failure annual review |
| 1223 | G58..11 | Cardiac failure |
| 9913 | 1O1..00 | Heart failure confirmed |
| 13189 | 662g.00 | New York Heart Association classification - class II |
| 5942 | G581.13 | Impaired left ventricular function |
| 72965 | 9Or3.00 | Heart failure monitoring first letter |
| 18853 | 662f.00 | New York Heart Association classification - class I |
| 19066 | 662h.00 | New York Heart Association classification - class III |
| 32911 | 9Or..00 | Heart failure monitoring administration |
| 19380 | 9Or0.00 | Heart failure review completed |
| 46672 | 388D.00 | New York Heart Assoc classification heart failure symptoms |
| 15058 | 14A6.00 | H/O: heart failure |
| 70619 | 8HHz.00 | Referral to heart failure exercise programme |
| 5255 | G581000 | Acute left ventricular failure |
| 83502 | 662p.00 | Heart failure 6 month review |
| 32671 | G580100 | Chronic congestive heart failure |
| 32945 | 8CL3.00 | Heart failure care plan discussed with patient |
| 9524 | G580.14 | Biventricular failure |
| 10079 | G580.12 | Right heart failure |
| 72386 | 9Or4.00 | Heart failure monitoring second letter |
| 60099 | 67D4.00 | Heart failure information given to patient |
| 27884 | G580200 | Decompensated cardiac failure |
| 26115 | 8HHb.00 | Referral to heart failure nurse |
| 103732 | 8CMK.00 | Has heart failure management plan |
| 17278 | G58z.12 | Cardiac failure NOS |
| 23707 | G580000 | Acute congestive heart failure |
| 95835 | 679X.00 | Heart failure education |
| 27964 | G582.00 | Acute heart failure |
| 10154 | G580.13 | Right ventricular failure |
| 48897 | 8HTL.00 | Referral to heart failure clinic |
| 26242 | ZRad.00 | New York Heart Assoc classification heart failure symptoms |
| 23481 | G581.11 | Asthma - cardiac |
| 110101 | 8I98.00 | Heart failure rehabilitation programme not available |
| 51214 | 662i.00 | New York Heart Association classification - class IV |
| 89650 | 9Or5.00 | Heart failure monitoring third letter |
| 106894 | 8IE1.00 | Referral to heart failure exercise programme declined |
| 101138 | G583.00 | Heart failure with normal ejection fraction |
| 43618 | G581.12 | Pulmonary oedema - acute |
| 32898 | 8H2S.00 | Admit heart failure emergency |
| 90193 | 9Or1.00 | Heart failure monitoring telephone invite |
| 83481 | 9N4w.00 | Did not attend heart failure clinic |
| 11424 | G580300 | Compensated cardiac failure |
| 104275 | G584.00 | Right ventricular failure |
| 106897 | G583.12 | Heart failure with preserved ejection fraction |
| 22262 | G1yz100 | Rheumatic left ventricular failure |
| 90192 | 9Or2.00 | Heart failure monitoring verbal invite |
| 12590 | G58z.11 | Weak heart |
| 46912 | 14AM.00 | H/O: Heart failure in last year |
| 106680 | 8HTL000 | Referral to rapid access heart failure clinic |
| 102585 | 8HgD.00 | Discharge from heart failure nurse service |
| 106198 | 661M500 | Heart failure self-management plan agreed |
| 94870 | G580400 | Congestive heart failure due to valvular disease |
| 106008 | 8CMW800 | Heart failure clinical pathway |
| 101137 | G583.11 | HFNEF - heart failure with normal ejection fraction |
| 95021 | 9N4s.00 | Did not attend practice nurse heart failure clinic |
| 21837 | G232.00 | Hypertensive heart&renal dis wth (congestive) heart failure |
| 105002 | 679W100 | Education about deteriorating heart failure |
| 69062 | 9N6T.00 | Referred by heart failure nurse specialist |
| 71235 | 8Hk0.00 | Referred to heart failure education group |
| 105542 | 8CeC.00 | Preferred place of care for next exacerbation heart failure |
| 107981 | 8IE0.00 | Referral to heart failure education group declined |
| 91288 | 8Hg8.00 | Discharge from practice nurse heart failure clinic |
| 66306 | SP11111 | Heart failure as a complication of care |
| 111428 | 2JZ..00 | On optimal heart failure therapy |
| 96799 | G5y4z00 | Post cardiac operation heart failure NOS |

**Table S2** Clinical codes used to identify heart failure in hospital records

| ICD code | Term |
| --- | --- |
| I50 | Heart failure |
| I50.0 | Congestive heart failure |
| I50.1 | Left ventricular failure |
| I50.9 | Heart failure, unspecified |
| I11.0 | Hypertensive heart disease with (congestive) heart failure |
| I13.0 | Hypertensive heart and renal disease with (congestive) heart failure |
| I13.2 | Hypertensive heart and renal disease with both (congestive) heart failure and renal failure |

**Table S3** Clinical codes used to identify natriuretic peptide tests

| **Medcode** | **Read Code** | **Read Term** |
| --- | --- | --- |
| 108309 | 44AV.00 | Serum N-terminal pro B-type natriuretic peptide conc |
| 27097 | 44AP.00 | Serum pro-brain natriuretic peptide level |
| 68734 | 4Q2B.00 | N terminal pro-brain natriuretic peptide level |
| 108338 | 44AX.00 | Serum B-type natriuretic peptide concentration |
| 14143 | 44AR.00 | Plasma B natriuretic peptide level |
| 108253 | 44AW.00 | Plasma N-terminal pro B-type natriuretic peptide conc |
| 40914 | 44AF.00 | Brain natriuretic peptide level |
| 14140 | 44AN.00 | Plasma pro-brain natriuretic peptide level |
